# Ceramic Nanofiltration Membranes: Creating Nanopores by Calcination of Atmospheric-Pressure Molecular Layer Deposition Grown Titanicone Layers

**DOI:** 10.3390/membranes15030086

**Published:** 2025-03-08

**Authors:** Harpreet Sondhi, Mingliang Chen, Michiel Pieter Nijboer, Arian Nijmeijer, Fred Roozeboom, Mikhael Bechelany, Alexey Kovalgin, Mieke Luiten-Olieman

**Affiliations:** 1 Inorganic Membranes, University of Twente, 7500 AE Enschede, The Netherlands; h.sondhi@utwente.nl (H.S.); m.chen-1@tudelft.nl (M.C.); m.p.nijboer@utwente.nl (M.P.N.); arian.nijmeijer@shell.com (A.N.); f.roozeboom@utwente.nl (F.R.); 2 Institut Européen des Membranes (IEM), École Nationale Supérieure de Chimie de Montpellier, Centre National de la Recherche Scientifique, Place Eugène Bataillon, UMR-5635 Université Montpellier, 34095 Montpellier, France; mikhael.bechelany@umontpellier.fr; 3 Functional Materials Group, Gulf University for Science and Technology, Mubarak Al-Abdullah 32093, Kuwait; 4 Integrated Devices and Systems, University of Twente, 7500 AE Enschede, The Netherlands; a.y.kovalgin@utwente.nl

**Keywords:** molecular layer deposition, titanicone, functionalization, ceramic membranes, nanopores, size-selective separation

## Abstract

Ceramic membrane technology, whether applied as a stand-alone separation technology or in combination with energy-intensive approaches like distillation, is a promising solution for lower energy alternatives with minimal carbon footprints. To improve the separation of solutes in the nanofiltration range from industrial wastewater streams, ceramic nanofiltration (NF) membranes with reproducible sub-nanometre pore sizes are required. To achieve this, the emerging technique of molecular layer deposition (MLD) is employed to develop ceramic NF membranes, and its efficiency and versatility make it a powerful tool for preparing uniform nanoscale high-porosity membranes. Our work, which involved vapor-phase titanium tetrachloride as a precursor and ethylene glycol as a co-reactant, followed by calcination in air at 350 °C, resulted in NF membranes with pore sizes (radii) around ~0.8 ± 0.1 nm and a demineralized water permeability of 13 ± 1 L·m^−2^·h^−1^·bar^−1^.The high-water flux with >90% rejection of polyethylene glycol molecules with a molecular size larger than 380 ± 6 Dalton indicates the efficiency of the MLD technique in membrane functionalization and size-selective separation processes, and its potential for industrial applications.

## 1. Introduction

Today, pharmaceutical, textile, and petrochemical industries use energy-intensive processes like distillation to purify, exchange, and recycle wastewater streams, which results in high operational costs [1,2,3]. Typically, polymeric membranes are used to treat these industrial water streams. However, for separation at elevated temperatures in combination with wastewater mixtures (sometimes contaminated with small concentrations of solvents) [4], these membranes are prone to swelling [5,6], making them inappropriate for high-temperature nanofiltration (NF) processes [2,7,8,9,10,11]. Based on the size-exclusion principle, the NF process utilizes pressure to separate contaminants from the wastewater streams [9,12,13,14].

In contrast, ceramic membranes remain unaffected under harsh process conditions (high temperatures, pressures, and wastewater mixtures). However, it is challenging to commercially functionalize ceramic membranes with a size-selective layer with nanopores of size < 2 nm and a molecular weight cut-off (MWCO) below 400 Da with existing state-of-the-art sol–gel processes while preserving identical properties (such as uniform pore size distribution and reproducibility).

Typically, two sol–gel process types exist: polymeric sol–gel (based on the chemistry of metal–organic precursors in organic solvents) and colloidal sol–gel (based on colloidal chemistry in aqueous media). The polymeric sol–gel method is most commonly used due to its inherent properties (preventing aggregation) [15]. However, it is not eco-friendly, as various hazardous solvents are used [16]. The colloidal sol–gel method is eco-friendly as it utilizes water as a solvent (non-toxic, less volatile, low-cost, and easily available) [16], which makes it the preferred choice for industrial-scale production. However, it is very challenging to fabricate titanium dioxide (TiO_2_) NF membranes due to high particle aggregation during the process. Compared to (alumina) Al_2_O_3_ and zirconia (ZrO_2_), TiO_2_ membranes are more chemically stable [4,6,15,16,17,18].

Thus, alternative techniques for functionalization at this atomic-size scale are specifically challenged by the membranes’ higher sensitivity to contamination where a dust-free process environment is required. In addition, variations in the sintering processes can lead to the aggregation of particles, leaving relatively large pore size distribution [13,19,20,21].

This study presents a promising solution to these challenges by using the molecular layer deposition (MLD) technique [22,23,24,25,26,27,28,29,30] to fabricate titanicone layers on ceramic supports followed by post-deposition calcination in air. MLD, a sub-type of atomic layer deposition (ALD) [25,26,28,29,31], is used to deposit hybrid inorganic–organic layers instead of just purely inorganic layers as grown with ALD with a controlled thickness and pre-selected organic constituents. These constituents typically decompose at specific temperatures (ranging from 250 up to 500 °C) [31,32,33] so that the MLD technique can be utilized to deposit titanicone layers from pre-selected organic co-reactants that will determine the nanopore size of the layers as grown. By careful selection of the co-reactant, one can tailor the functionalities such as pore size, pore size distribution, and surface hydrophobicity [34].

This approach allows us to reproducibly manufacture and functionalize ceramic membranes for nanofiltration (NF) applications in the purification of wastewater fluxes with size-selective separation. It could also significantly improve the efficiency and cost-effectiveness of ceramic nanofiltration processes.

## 2. Experimental Methods

### 2.1. Materials

Alpha alumina (α-Al_2_O_3_) ceramic supports (CoorsTek, Uden, Netherlands, AD-998 with average pore size; diameter of 20 nm [35]), silicon (100) wafer coupons (SUMCO, Tokyo, Japan), ≥ 97% titanium tetrachloride (TiCl_4_), and 99.8% ethylene glycol (EG, CH_2_OH)_2_) were procured from Sigma-Aldrich, Amsterdam, Netherlands.

### 2.2. Methodology: Atmospheric-Pressure MLD (AP-MLD)

Tubular membrane supports (α-Al_2_O_3_, 1 mm thickness, 100 mm in length, inner diameter of 70 mm) were used as a substrate for layer deposition. A custom-built atmospheric-pressure MLD reactor (AP-MLD) was utilized. For general fundamentals of MLD, see Figure S1 (Supplementary Material (SM)), and for more details on AP-MLD, see also ref. [36]. AP-MLD provides coatings with high quality comparable to those deposited in vacuum-based systems. No vacuum is needed, thus facilitating scaling up at an industrial scale [36,37].

### 2.3. Preparation of Titanicone Layers Inside Tubular α-Al_2_O_3_

To fabricate the titanicone layers, titanium tetrachloride (TiCl_4_) was selected as a precursor and ethylene glycol (EG) as a co-reactant. TiCl_4_ and EG were selected on criteria like their size, high bifunctional reactivity, and cost, as has been widely reported in MLD studies [31,38]. As described in Section S2 (SM), a similar approach was used for the titanicone layer formation on the ceramic support. The precursor and co-reactant were supplied as vapours from canisters held at room temperature, as listed in Table S1 (SM). Nitrogen was used as an inert purging gas. The pulse times used were 1 s for TiCl_4_, 2 s for EG, and 150 s for N_2_ purging, where a relatively longer purge time was used to ensure the presence of strictly one chemical at a time. Due to its monomeric and bifunctional nature, EG can react as a functionalizing or cross-linking agent to form a poly(titanium ethylene glycol) polymer [38,39]. Based on these reports, MLD was conducted at a set temperature of 125 °C. First, a TiCl_4_ pulse was introduced for adsorption on the inside surface of the tubular support. After a subsequent N_2_ purge step, the EG pulse was introduced, which readily reacted to form a titanicone monolayer (see Figure S2 (SM)). A total of 450 MLD cycles were used to prepare a layer with a thickness of 20 nm. After the layer deposition, it was calcined in air at 350 °C. Calcination leads to the formation of calcined titanicone hybrid layers where the organic parts partly or fully decompose, yielding a uniform pore-size distribution. It is known from the literature that temperature is crucial in layer functionalization and tailoring properties [32,34].

### 2.4. Characterization

A J.A.Woollam (M-2000) spectroscopic ellipsometer [40] was applied to measure the thickness of the titanicone layer grown on the planar silicon wafer and calculate the growth per cycle (Å). A Zeiss Merlin high-resolution scanning electron microscopy (HR-SEM) system (field emission source with 1.2 nm resolution) was used for cross-section imaging and energy dispersive X-ray (EDX) spectroscopy for elemental analysis. A PerkinElmer-Spectrum Two FT-IR spectrophotometer within the 400–4000 cm^−1^ wavenumber range was used for bulk composition analysis. Nanopore size (radius) and rejection rate (based on molecular size) measurement studies were conducted using an IKTS Fraunhofer (Dresden, Germany) permporometry (PPM) setup and a home-built molecular weight cut-off (MWCO) (University of Twente, Enschede, Netherlands) tool. Five replicates were performed for each calcined titanicone hybrid layer membrane, from which the standard deviation was calculated and plotted as error bars. The size of the analyte molecules was measured with a gel permeation chromatography (GPC) and size exclusion chromatography (SEC) system (Agilent Technologies, Middleburg, Netherlands, 1200/1260 Infinity GPC/SEC series) [41].

#### 2.4.1. In-Line Gas Permeance Study of Tubular α-Al_2_O_3_ Membrane

The AP-MLD reactor was used to measure the in-line gas permeance through the titanicone layers deposited inside the tubular α-Al_2_O_3_ membrane to monitor the layer growth and to confirm that the top surface over the pores was sealed off by the MLD titanicone layers before calcination.

#### 2.4.2. Titanicone Layer Thickness Study on Planar Silicon Substrates

The layer thickness is critical in tuning the nanopore sizes and solvent permeation after calcination. First, to achieve titanicone layers with a certain thickness (set at 20 nm) to completely cover the pores on the surface of the support, the number of cycles was adjusted based on the data listed in Table S1. Spectroscopic ellipsometry was used to verify layer thicknesses (as measured for films grown on planar Si-substrates, described in detail in the SM (Section S2)).

#### 2.4.3. Degree of Porosity and Elemental Distribution of Calcined Titanicone Hybrid Layers on Tubular α-Al_2_O_3_

An HR-SEM analysis in cross-section mode was used to investigate the degree of porosity and uniform elemental distribution of the titanium inside the MLD grown titanicone layers. An EDX analysis was used to map the elemental titanium present across the tubular membrane depth.

#### 2.4.4. Layer Composition on Tubular α-Al_2_O_3_

To determine the characteristic vibration modes of carbon- and titanium-containing functional groups, FTIR in transmission mode was used to investigate the bulk composition of the freshly deposited titanicone and post-deposition calcined titanicone hybrid layers, and the pristine α-Al_2_O_3_ support.

#### 2.4.5. Pore Size and Distribution, and Size-Selective Separation for Calcined Titanicone Hybrid Layers on Tubular α-Al_2_O_3_

Permporometry was used to measure the size of active pores of the calcined titanicone hybrid layers and their distribution on tubular α-Al_2_O_3_ membranes using water as a condensable vapor (described in detail in the SM (Section S3). A closed-loop filtration setup at a pressure of 10 bar was used to measure the MWCO of the membranes. The retention of an aqueous solution containing 1 wt.-% of polyethylene glycol molecules (PEG) with average molecular weights (M_w_ of 200, 300, 400, 600, 1000, 1300, 1500, and 2000 g.mol^−1^) was measured. The samples from the feed and permeate were measured using gel permeation chromatography for the size-selective separations.

## 3. Results and Discussion

### 3.1. Degree of Porosity, Elemental Distribution, and Composition of Calcined Titanicone Hybrid Layers on Tubular α-Al_2_O_3_ Analysis

**Figure 1:**
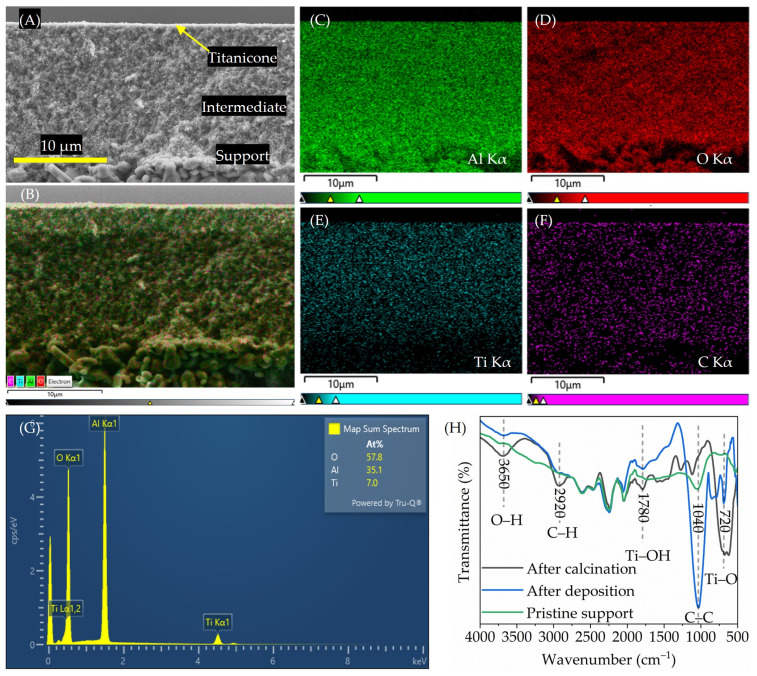
(**A**,**B**) Tilted view cross-sectional HR-SEM images of a nominally 20 nm thick TiCl_4_-EG titanicone layer grown on the tubular α-Al_2_O_3_ substrate and subsequently annealed at 350 °C. Elemental mappings of constituting elements altogether (**B**) and individually per element: (**C**) Al (Kα), (**D**) O (Kα), (**E**) Ti (Kα), and (**F**) C (Kα). (**G**) EDX spectrum of a calcined titanicone hybrid layer. (**H**) FTIR spectrum of pristine α-Al_2_O_3_ (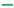), and α-Al_2_O_3_ coated with titanicone layers deposited at 125 °C (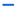) and after calcination at 350 °C (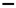). Vertical scale bars (**A**–**F**) 10 µm.

Figure 1A,B shows the HR-SEM cross-sectional micrographs of a typical calcined titanicone hybrid layer over the ceramic support surface. The three zones (support, intermediate layer, and titanicone layer) in the micrograph (Figure 1A) have different electron backscattering properties, and clear distinctions can be made. Also, due to the porous nature of the substrate and the calcined titanicone hybrid layers, it has been indicated in the HR-SEM micrographs that there is a change in the porosity of the membrane. Figure 1B–F shows the elemental mappings, and Figure 1G shows the result of a characteristic line scan investigated using EDX. It can be concluded that elemental titanium is incorporated into the depth of the MLD-grown layer on the ceramic support. Furthermore, after calcination, the remaining carbon due to inter- and intra-molecular (de-)bonding results in uniformly distributed carbon species depth-wise through the layer, as shown in the EDX spectrum for C-Kα in Figure 1F. FTIR measurements were carried out for the bulk composition analysis, supporting the EDX findings, as shown in Figure 1H. The FTIR spectra indicate a significant reduction in the amount of carbon inside the layer after calcination, and Figure 1F shows the distribution of the remaining carbon species in detail. The characteristic vibrations of covalent bonds of C-H at 2920 cm^−1^ correspond to the remaining carbon species, and Ti-OH and Ti-O at 1780 cm^−1^ and 740 cm^−1^ [42,43] further confirm the formation of a calcined titanicone hybrid layers with elemental titanium in it. It also confirms our hypothesis that the amount of carbon species reduces drastically in the layer after calcination, thus decomposing the organic species.

### 3.2. In-Line Gas Permeance Analysis

**Figure 2:**
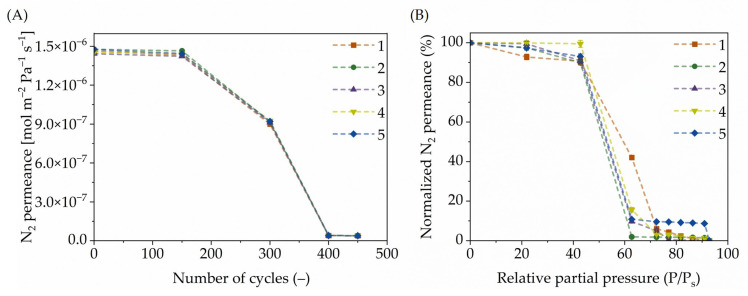
N_2_ permeance measurements of five identically grown, nominally 20 nm thick titanicone hybrid layer membranes. (**A**) Before calcination, N_2_ permeance measured in-line as a function of number of growth cycles. (**B**) After calcination at 350 °C, N_2_ permeance normalized as a function of the partial pressure of water vapor. The membranes are labelled as 1 (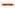), 2 (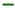), 3 (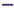), 4 (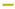), and 5 (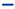).

In-line gas permeance measurements were conducted to monitor the layer growth and to confirm the complete sealing off of the top surface over the pores inside the α-Al_2_O_3_ tubular support by the MLD of the titanicone layers. Nitrogen gas permeance was measured regularly from the start until the end of deposition using intermittent pressure-drop tests (for further details, see also ref. [36]). As shown in Figure 2A, after 400 cycles of MLD, the nitrogen permeance dropped drastically, indicating the effectiveness of 400–450 MLD cycles in growing dense titanicone layers over the pores of the α-Al_2_O_3_ tubular support from TiCl_4_ and EG using MLD. Importantly, the high reproducibility of this process is underscored by the fact that the data points for all five membranes overlap after 150 cycles, demonstrating the rapid and reliable surface coverage due to MLD, resulting in dense titanicone layers over the pores.

### 3.3. Permporometry Analysis Using Water as a Condensable Vapor

Permporometry measurements were conducted using water as a condensable vapor to measure the pore size and distribution. In Figure 2B, the nitrogen permeance through the membrane is shown. The average pore size (radius) can be deduced from the pore size distribution curve, typically defined at 50 % permeance; it is sharp and centred around 0.8 nm (as shown in Figure S3B). One crucial parameter is the reproducibility of the measurement using five identically prepared membranes. As shown in Figure 2B, all membranes, labelled 1, 2, 3, 4, and 5, show a similar pore-size distribution curve trend. These membranes were calcined at 350 °C, with similar decomposition behaviour of their organic parts (EG), thus generating nanopores that can act as the transport channel for solvents. The literature reports that, when water vapor is used as a condensable medium during PPM measurements, it does not affect the adsorption layers. This means that compared to alcohols (methanol and ethanol), carbon tetrachloride, and hexane (which are relatively larger molecules), water, due to its small size and without alkyl groups, does not plug the pores (no Van der Waals interactions). Depending on the pore shape factor [17] of the support, water does not affect nitrogen permeation [20,44,45,46,47]. The specific enthalpy of vaporization is very high for water compared to alcohols. This makes water easy to adsorb between 0 and 10 °C, while alcohols require temperatures below 0 °C (for further details, see [48] and Section S3 (SM)). Thus, the pore sizes measured are relatively closer to the actual pore size of the calcined titanicone hybrid layer membranes.

### 3.4. Molecular Weight Cut-Off Analysis Using PEG as Solute Molecules and Water Permeability

**Figure 3:**
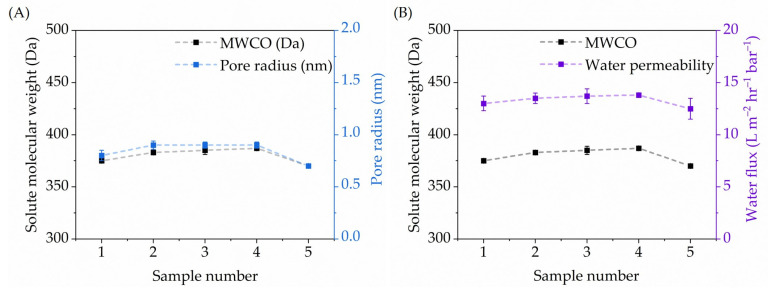
Molecular weight-cut-off values (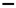) with respect to (**A**) pore radius (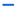) and (**B**) water flux (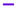) of calcined titanicone hybrid layer membranes, calcined at 350 °C.

The MWCO measurements, in combination with gel permeation chromatography, were conducted to determine the cut-off of the membranes. This was achieved by conducting separation experiments using standard polyethylene glycol molecules as solutes with different molecular weights with a defined rejection of 90 % (see [49] and Figure S3C) as measured using gel permeation chromatography. Figure 3A presents the retention rate curves of the five measured calcined titanicone hybrid layer membranes. Table 1 provides the MWCO data of all five membranes and the average pore size (radius) measured by PPM. The membrane MWCO, an essential parameter for membrane transport mechanisms, allows us to select the rejection characteristics as an indicator to compare the differences in membrane performance. Table 1 summarizes the results of the measurements by PPM, MWCO, and demineralized water permeability; see also Figure 3. All pore sizes (radii) are closely centred around ~0.8 nm. This is in qualitative agreement with the PPM measurements conducted on titania layers with sub-nanometre pore size as formed by sol–gel preparation [12] with a pore size (radius) ~0.9 nm or smaller, as estimated from the MWCO values for polyethylene glycol (PEG) as a solute with M_w_ = 400 g mol^−1^.

This remarkably high reproducibility also holds for MWCO (~380 Da) and for the demineralized water permeability (~13 L·m^−2^·h^−1^·bar^−1^), all having minimal standard deviation. This finding is particularly significant because, for the similar MWCO range, MLD-prepared membranes show a higher water flux, i.e., twice the throughput for the same filtration efficiency compared to that of state-of-the-art commercial polymeric membranes [50].

### 3.5. Highlights of Experimental Results and Outlook

Functionalizing ceramic nanofiltration membranes using atmospheric-pressure MLD;

MLD of titanicone layers from titanium tetrachloride with ethylene glycol;

Post-deposition air calcination creates sub-nanometre range pore sizes;

Option for the manufacture of membranes with improved performance and size-selectivity.

The objective was to accurately follow the functional properties of the titanicone layers used to functionalize ceramic membranes on α-Al_2_O_3_ support and their subsequent calcination. Post-deposition calcination was carried out in an air environment at 350 °C to understand the influence of temperature on the functional groups (e.g., the nature and the relative amounts of carbon species) contained by the hybrid layers. A complete sealing off of the top surface over the pores for all titanicone layers was confirmed with in-line gas permeance measurements. Using a combination of ex situ HR-SEM and FTIR measurements, the elemental distribution of titanium and change in functional groups for the MLD-grown titanicone layers on bare support before and after calcination could be correlated. FTIR studies also revealed pronounced characteristic vibrations for the calcined titanicone hybrid layer with respect to the pristine support. HR-SEM further confirmed the uniform distribution of elemental titanium into the depth of the calcined titanicone hybrid layer grown with MLD on the α-Al_2_O_3_ ceramic support. As reported in the literature [14,32], EG molecules decompose partly and fully at 250 °C and 350 °C, respectively, during calcination in air. Thus, calcination causes the carbon species in the bulk of the titanicone hybrid layer to decompose, as observed in FTIR measurements.

**Figure 4:**
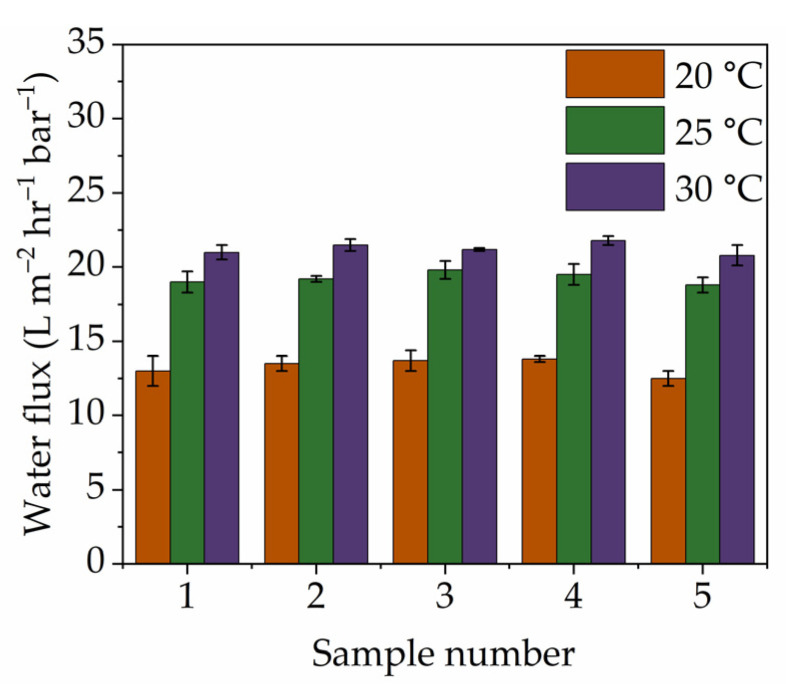
Repeated filtration cycle permeability for demineralized water as a function of three different temperatures, 20 °C (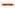), 25 °C (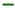), and 30 °C (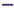), for calcined titanicone hybrid layer membranes.

Further performance testing of the calcined titanicone hybrid layer membranes was performed using PPM and MWCO. Five membranes were used, and each one was measured five times for long-term stability, replication, and statistical analysis (see Figure 4) under similar operational conditions for pore size and at three different temperatures (20, 25, and 30 °C) for demineralized water permeability measurements. Each membrane had a runtime of 15 h (5 h per temperature) in contact with water. PPM measurements further confirmed the sub-nanometre range pore sizes and narrow pore-size distribution of the calcined titanicone hybrid layers. MWCO studies also revealed the active rejection of solutes (PEG) from solvent (water), with sizes above 390 Da and high water permeability. Compared with the relevant literature, where commercial polymeric membranes (i.e., NF200) [50] were used as the state of the art, as listed in Table 2, the NF membranes prepared in this work exhibited high water permeability at lower operational pressures. Table 2 summarizes the results of the measurements by MWCO and water permeability. The NF200 has a range of MWCO values, which can be compared with the present study. As shown in Figure 4, a statistical analysis was carried out by measuring the permeability for demineralized water through calcined titanicone hybrid layer membranes at 20, 25, and 30 °C (similar to NF200). This test clearly shows that the throughput is twice for each temperature, respectively. These membranes were efficient also after five repetitive filtration cycles (see Figure 4) at these temperatures, thus underpinning the longer-term stability and functional properties of the size-selective layer prepared using MLD-grown titanicone layers.

Thus, functionalized ceramic membranes, under harsh conditions, hold the potential to significantly cut the energy consumption needed for nanofiltration based on the separation of molecules by size exclusion. The ability to tailor the porous support surface may facilitate the synthesis of ceramic solvent-tolerant nanofiltration (STNF) membranes with sub-nanometre range pore sizes.

Industrial process streams can also be water-based and rich in organic solvents [51]. Thus, specialized STNF membranes with hydrophobic surfaces are required to efficiently remove solutes from organic solvent streams. Currently, in tuning the surface hydrophobicity of large surface area substrates, the state-of-the-art silanization process solutions are still challenged by the limited control over the key process parameters (reagent concentration, reaction time, and temperature) [52,53]. As per the literature [34] on planar ceramic supports, the MLD technique can be employed to tune the surface hydrophobicity. We envisage that expanding the research further into porous multilayer ceramic membranes (e.g., tubes, etc.) with a focus on long-term stability and anti-fouling measurements will generate new options in low-cost large-scale organo-solvent nanofiltration technology.

## 4. Conclusions

Titanicone membrane networks were developed for nanofiltration (NF) with a sharp cut-off, capable of actively rejecting solute molecules above 380 ± 6 Da with a rejection rate of >90%. To this end, tubular α-Al_2_O_3_ ceramic supports were pre-functionalized by molecular layer deposition (MLD). After subsequent calcination at 350 °C in air, surface layers were obtained with sub-nanometre pore sizes (radii) centred around ~0.8 ± 0.1 nm and a demineralized water permeability of 13 ± 1 L·m^−2^·h^−1^·bar^−1^. This permeability is twice the throughput compared to the commercially available polymeric membranes with similar filtration efficiency.

The careful selection of the organic co-reactant chemicals for MLD is essential in refining the desired membrane functionalities. Our results demonstrate the effectiveness of the MLD approach and highlight, in particular, the potential of titanicone layers deposited from TiCl_4_ and organic co-reactant EG to coat ceramic oxide membranes.

The implication of this highly reproducible, solvent-free (i.e., dry-process) approach for tuning the pore size on an atomic-size scale with AP-MLD opens up avenues for developing industrial-scale nanofiltration membranes with highly improved performance and selectivity.

## Figures and Tables

**Table 1:** Overview of the measured characteristics of the calcined titanicone hybrid layers on α-Al_2_O_3_.

Sample	Pore Radius (nm)	MWCO (Dalton)	Water Flux ( L·m^−2^·h^−1^·bar^−1^)
	(Standard dev.~0.1)	(Standard dev.~6)	(Standard dev.~1)
1	0.8	375	13
2	0.9	383	13.5
3	0.9	385	13.7
4	0.9	386	13.8
5	0.7	370	12.5

**Table 2:** Overview of the filtration characteristics of the NF200 (commercial) and calcined titanicone hybrid layer membrane on α-Al_2_O_3_.

Membrane Type	Temperature (°C)	Pressure (bar)	MWCO (Da)	Water flux (L·m^−2^·h^−1^·bar^−1^)
NF200 [50]	20	20	300–360	7.7
	25	20	-	8.65
	30	20	-	9.71
Calcined titanicone hybrid layer	20 25 30	9 9 9	380 ± 6 - -	13 ± 1 19 ± 0.7 21 ± 0.5

## Data Availability

Data are contained within the article.

## Supplementary Materials

The following supporting information can be downloaded at https://www.mdpi.com/article/10.3390/membranes15030086/s1, Section S1:Molecular layer deposition; S2: Titanicone layer on silicon (100) wafer coupons (TiO_2_-ALD coated-silicon); S3: Permporometry and Molecular weight cut-off measurements; Figure S1: Schematic of MLD cycling for the preparation of one monolayer of hybrid material; Figure S2: Schematic representation of fabrication of one ‘titanicone’ monolayer formed upon MLD of TiCl_4_ and ethylene glycol on an α-Al_2_O_3_ ceramic support and the subsequent formation of nanoporous calcined hybrid layers on the ceramic support; Figure S3: Characteristics of measurement spectra of calcined hybrid layers membranes. Dimensionless N_2_ permeance (A) as a function of relative partial pressure and (B) as a function of pore radius (nm). (C) Molecular weight cut-off with solute (PEG) rejection of 90 %; Table S1: Overview of the process parameters for MLD growth at 125 °C.
