# Supplementary material for: Ceramic Nanofiltration Membranes: Creating Nanopores by Calcination of Atmospheric-Pressure Molecular Layer Deposition Grown Titanicone Layers
Source: Membranes (Basel). 2025 Mar 8;15(3):86. doi: 10.3390/membranes15030086 (PMC11943934; doi:10.3390/membranes15030086)
Supplement: Supplementary file 1 [file membranes-15-00086-s001.zip › membranes-3490338-supplementary.pdf]

Supplementary Materials

S1. Molecular layer deposition

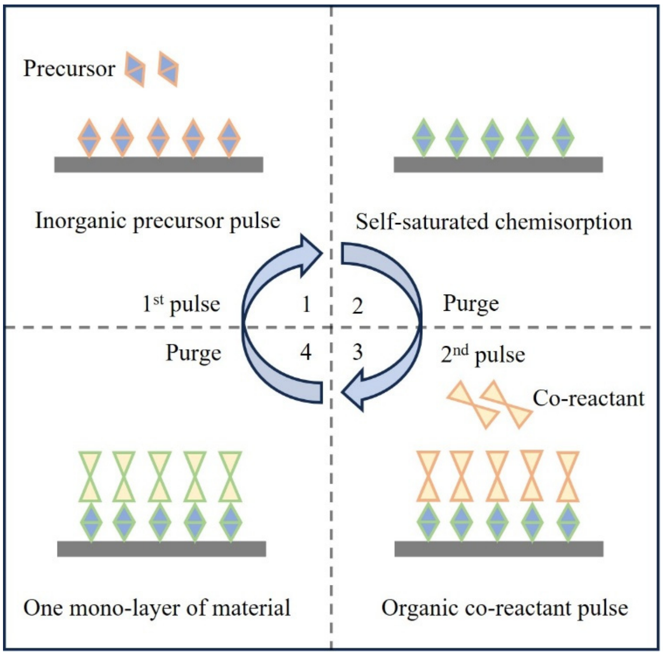

Figure S1: Schematic of MLD cycling for the preparation of one monolayer of hybrid material.

S2. Titanicone layer on silicon (100) wafer coupons (TiO_2-ALD coated-silicon)

Silicon (100) with a thickness of 525 µm, p-type, low B-doped with 10^{15} B-atoms.cm^{-3} (resistivity of 5-10 Ω.cm) and coupon size 10 × 0.7 cm^2 were used to measure the thickness of the deposited layers. From the thickness one can calculate the growth per cycle (GPC) for the precursor and co-reactant. From the GPC one can deduce the layer thickness in Angstrom (Å) as deposited during each cycle of MLD. Two reactants were used to synthesize the titanicone deposit: $TiCl_4$ and ethylene glycol (EG). The ALD cycle starts with introducing $TiCl_4$ vapor into the reaction chamber, which a hydroxylated surface will adsorb; as shown in Figure S2. Then, once the first surface reaction is completed, EG is introduced. Inert gas nitrogen (N_2) is purged after each pulse to remove all unreacted species and byproducts. A simplified reaction scheme for the surface half-reactions during MLD can be written as:

╟OH + $TiCl_4 \rightarrow$ ╠O_2-Ti-Cl_2 + 2 HCl (1)

╟O-$TiCl_3$ + $C_2H_6O_2 \rightarrow$ ╟O-Ti-$C_2H_3O_2$ + 3 HCl (2)

╟, ╠ Symbols indicate monodentate and bi-dentate adsorption to surface sites, respectively.

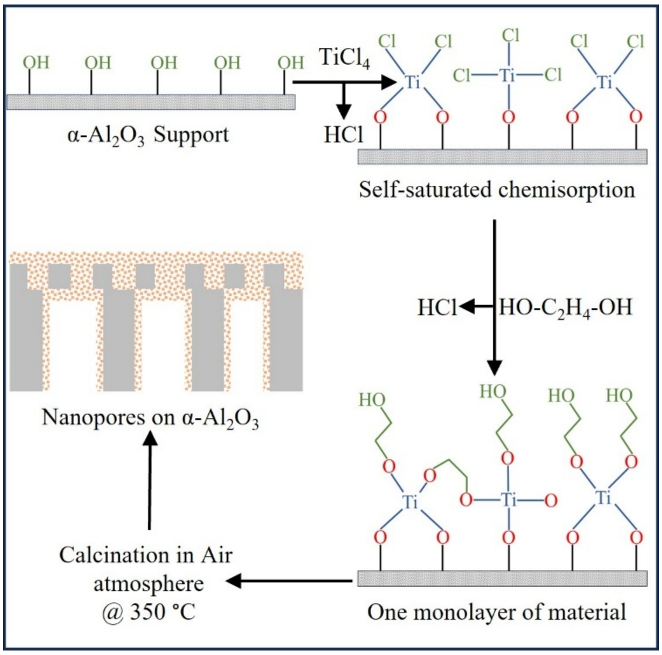

Figure S2: Schematic representation of fabrication of one 'titanicone' monolayer formed upon MLD of $TiCl_4$ and ethylene glycol on an α-Al_2O_3 ceramic support and the subsequent formation of nanoporous calcined hybrid layers on the ceramic support.

Table S1: Overview of the process parameters for MLD growth at 125 °C.

Reactant	Pulse time (s)	N_2 purge time (s)	Number of cycles	GPC (nm)
Titanium tetrachloride	1	150	450	0.05
Ethylene glycol	2	150		

S3. Permporometry and Molecular weight cut-off measurements

As shown in Fig. S3(A), this typical plot of the dimensionless N_2 permeance vs. the relative partial pressure can be divided into three parts. When the pressure is below 50, a slight decrease in the N_2 flux through the membrane is observed, but not too much. This could be attributed to the region where all pores with pore radius, r_p are readily available for transport and are empty. The Kelvin equation (3) is used to calculate the pore radius. At a relative partial pressure between 50 and 90, as the pressure increases, the N_2 flux decreases resulting in more adsorption of water vapors. In the interval $90 < p_r < 100$, pores are assumed to be blocked with condensate (water), and diffusion of free gas is obstructed. It is the point where the capillary condensation process, meaning multilayer adsorption, takes place, which results in the gradual filling of the pores with the condensate. Furthermore, as shown in Fig. S3(B), the dimensionless N_2 permeance increases with decreasing pore size.

$$RTln\left(\frac{P}{P_S}\right) = 2v\frac{\sigma\, cos\theta}{r_p} \quad (3)$$

The measurements are conducted at a temperature range between 4 – 6 °C. Ethanol and methanol have high vapor pressures compared to water, but water has the highest specific enthalpy of vaporization: water (2477 kJ/kg), ethanol (774 kJ/kg), and methanol (1126 kJ/kg) at 10 °C. Chilling ethanol and methanol above 0 °C is challenging, which is not the case with water. Due to this reason, water can form a thin adsorption layer over the pores. At the same

time, for other condensable media (alcohols), increasing the number of cycles yields pore plugging before the adsorption layer formation.

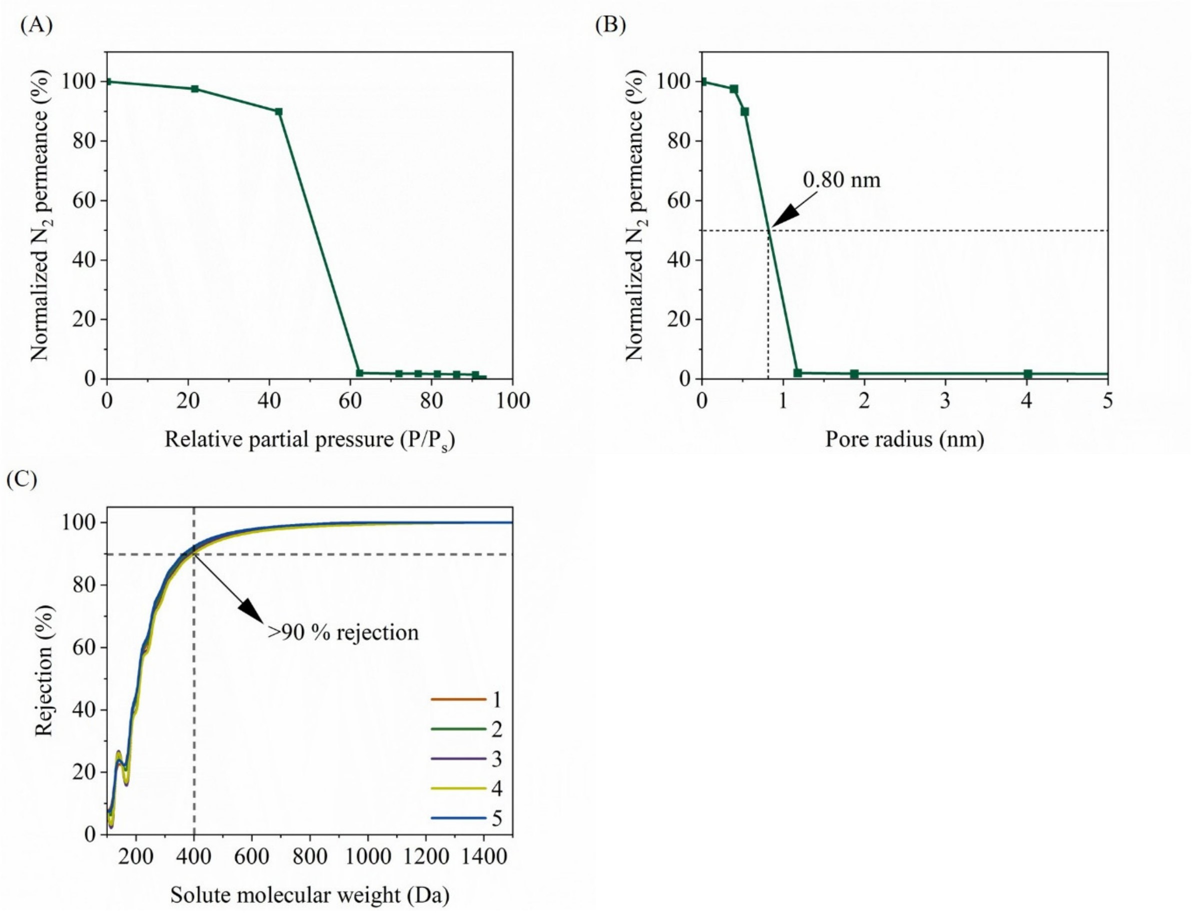

Figure S3: Characteristics of measurement spectra of calcined hybrid layers membranes. Dimensionless N_2 permeance (A) as a function of relative partial pressure and (B) as a function of pore radius (nm). (C) Molecular weight cut-off with solute (PEG) rejection of 90 %.
